# Pose estimation and motion analysis of ski jumpers based on ECA-HRNet

**DOI:** 10.1038/s41598-023-32893-x

**Published:** 2023-04-15

**Authors:** Wenxia Bao, Tao Niu, Nian Wang, Xianjun Yang

**Affiliations:** 1 School of Electronics and Information Engineering, Anhui University, Hefei, 230601 Anhui China; 2 Hefei Institutes of Physical Science, Chinese Academy of Sciences, Hefei, 230031 Anhui China

**Keywords:** Engineering, Electrical and electronic engineering

## Abstract

Ski jumping is a high-speed sport, which makes it difficult to accurately analyze the technical motion in a subjective way. To solve this problem, we propose an image-based pose estimation method for analyzing the motion of ski jumpers. First, an image keypoint dataset of ski jumpers (KDSJ) was constructed. Next, in order to improve the precision of ski jumper pose estimation, an efficient channel attention (ECA) module was embedded in the residual structures of a high-resolution network (HRNet) to fuse more useful feature information. At the training stage, we used a transfer learning method which involved pre-training on the Common Objection in Context (COCO2017) to obtain feature knowledge from the COCO2017 for using in the task of ski jumper pose estimation. Finally, the detected keypoints of the ski jumpers were used to analyze the motion characteristics, using hip and knee angles over time (frames) as an example. Our experimental results showed that the proposed ECA-HRNet achieved the average precision of 73.4% on the COCO2017 test-dev set and the average precision of 86.4% on the KDSJ test set using the ground truth bounding boxes. These research results can provide guidance for auxiliary training and motion evaluation of ski jumpers.

## Introduction

Ski jumping is a Winter Olympic sport in which it is challenging to establish effective measurement and kinematic analysis, due to the complex outdoor environments, high speeds, wide range of motion, and safety^1,2^ and health^3^ considerations. The traditional manual image annotation method, in which kinematic analysis software is used to annotate each keypoint of an athlete in continuous images and the annotated data are then used to calculate kinematic elements, is time-consuming and labor-intensive. However, it can be used to collect kinematic data without hindering the motion of the athlete when participating in regular training or competition, and has value as a method of motion analysis. Current methods of motion analysis primarily rely on wearable devices^4,5^ (for example based on inertial measurement units). This method provides accurate data, but the devices are cumbersome to wear and can affect an athlete's performance to some extent. In recent years, computer vision techniques have evolved rapidly, and machine learning has been used to train a human pose estimation model to detect human keypoints from images. This approach can replace the manual annotation process, and can significantly reduce the data processing time needed for technicians to manually annotate athletes and then analyze the motion (also known as manual digitization^6^). Hence, the task of ski jumper pose estimation using computer vision techniques has strong research significance and application value.

Currently, two types of deep learning technique are widely used in vision-based human pose estimation. The first category is top-down methods, in which object detection of all human bodies in the image is carried out, and each human body is then cropped into single images. Single-person pose estimation is then applied to each cropped image. Classical models include Hourglass^7^, CPN^8^, SimpleBaseline^9^, HRNet^10^, and others. In particular, there are two main methods for human object detection for images: two-stage (i.e., region proposal) methods and one-stage (i.e., regression) methods. In a two-stage method, a region is first generated called the region proposal (RP) box, and is fed into the network to extract the features. Then, the category of each proposal box is predicted and optimized. The most common models include R-CNN^11^, Fast R-CNN^12^, and Faster R-CNN^13^. The one-stage approach is an end-to-end method that simultaneously predicts the class and location of the object after extracting the features in the network, without the need for a suggested region. Commonly used models are SSD^14^ and YOLO^15^. Of these two methods, the two-stage approach has higher accuracy, while one-stage models have significantly higher detection speed and efficiency.

The second category of human pose estimation methods are based on bottom-up approaches. These methods first detect keypoints for all of the people in an image and then cluster them to different individuals by post-processing, thus eliminating the need for object detection networks, such as OpenPose^16^, HigherHRNet^17^, etc. A comparison of the two human pose estimation methods shows that the first type is usually more accurate, whereas the second gives speeds that are closer to real time.

In recent years, human pose estimation based on computer vision technology has been gradually applied to sports analysis and performance prediction. Fani et al.^18^ proposed an improved hourglass network for pose estimation of hockey players, and demonstrated the effectiveness of their method for automatic action recognition in the hockey field. Huang^19^ used OpenPose^15^ as a human keypoint detection model to perform human pose recognition on two-dimensional (2D) image signals. The detected keypoint data were converted to clinical test indices to correct sports pose with the aim of reducing athletic injuries and the difficulty of traditional manual angle measurement at the same time. Fei Lei et al.^20^ enhanced the precision of human keypoint detection on a public dataset by improving a stacked hourglass network, and applied it to pose estimation in complex environments (images of single divers). Erwin et al.^21^ employed a residual convolutional neural network to estimate the continuous 2D upper-body pose of a table tennis player. A recurrent long short-term memory (LSTM) network was then used to learn the player's serve motion and to predict the landing point of the table tennis ball.

The human pose estimation methods described above have achieved some significant research results when applied to sports. However, the high-speed motion means that images of ski jumping experience a motion blurring phenomenon, which makes estimating the athletes' poses more challenging. To date, very few studies have applied this technique to the field of ski jumping or other winter sports (such as freestyle skiing, etc.) to analyze the motion of skiers. Nam et al.^22^ proposed a hybrid framework that combines HigherHRNet^17^ in human pose estimation method with model-based force calculation to predict ski jumping forces from recorded motion videos. Ludwig et al.^23^ used Mask R-CNN^24^ in human pose estimation method for pose estimation and skis detection to evaluate the flight parameters for ski jumpers. Within the angle threshold of 5 degrees, 98.0% of the flight parameters could be correctly identified. Elfmark^25^ used a differential GNSS and a pose estimation system based on EfficientPose^26^ to measure kinematic and kinetic parameters from the in-run phase to the landing phase for 16 national ski jumpers, and assessed the consistency of the two methods. Furthermore, the study demonstrated the feasibility of applying both methods to analyze the kinematic and kinetic characteristics of ski jumping practice.

To achieve a more accurate motion analysis, we employ a top-down method of human pose estimation in this study to estimate the keypoints of the ski jumpers. Since YOLOv3^27^ represented an improvement on YOLO and YOLOv2^28^, with greatly improved detection precision, it has been applied to many engineering applications. Hence, YOLOv3 was selected for the object detection network in this study. HRNet, which was proposed by Sun et al.^10^, is a top-down method with two significant advantages over other human pose estimation methods: the use of parallel connection and repetitive multi-scale feature information fusion. These two advantages are important for ski jumping images with motion blur. We therefore improve HRNet for ski jumper pose estimation. The main contributions of this work are as follows:

i. Using video data obtained by high-speed cameras, we constructed a dataset of keypoint images of ski jumpers called KDSJ, which contained images representing the five phases of ski jumping (in-run, take-off, early flight, stable flight, and landing). The keypoints of the ski jumpers in these images were labeled under the guidance of an experienced technician, meaning that this dataset could be effectively used to test the performance of ski jumper pose estimation methods.
ii. A ski jumper keypoint detection method called ECA-HRNet was proposed. An ECA^29^ module was embedded in the multiscale feature extraction process of HRNet to break the independence between the keypoints of the athlete. It can link the connectivity between athlete keypoints in blurred images through the interaction of local cross-channel information, thus improving the precision of athlete keypoint detection.
iii. A transfer learning strategy was used in which feature knowledge from the public dataset COCO2017 was transferred to the task of ski jumping. The ECA-HRNet model was then fine-tuned using KDSJ to obtain the model with the best precision. At the same time, transfer learning improved the generalization ability of the network, speeding up the training efficiency and preventing overfitting.
iv. By detecting the keypoint data of ski jumpers from continuous images, we calculated trend graphs for the hip and knee angles over time (frames) and made training recommendations.

## Data acquisition and processing

**Figure 1:**
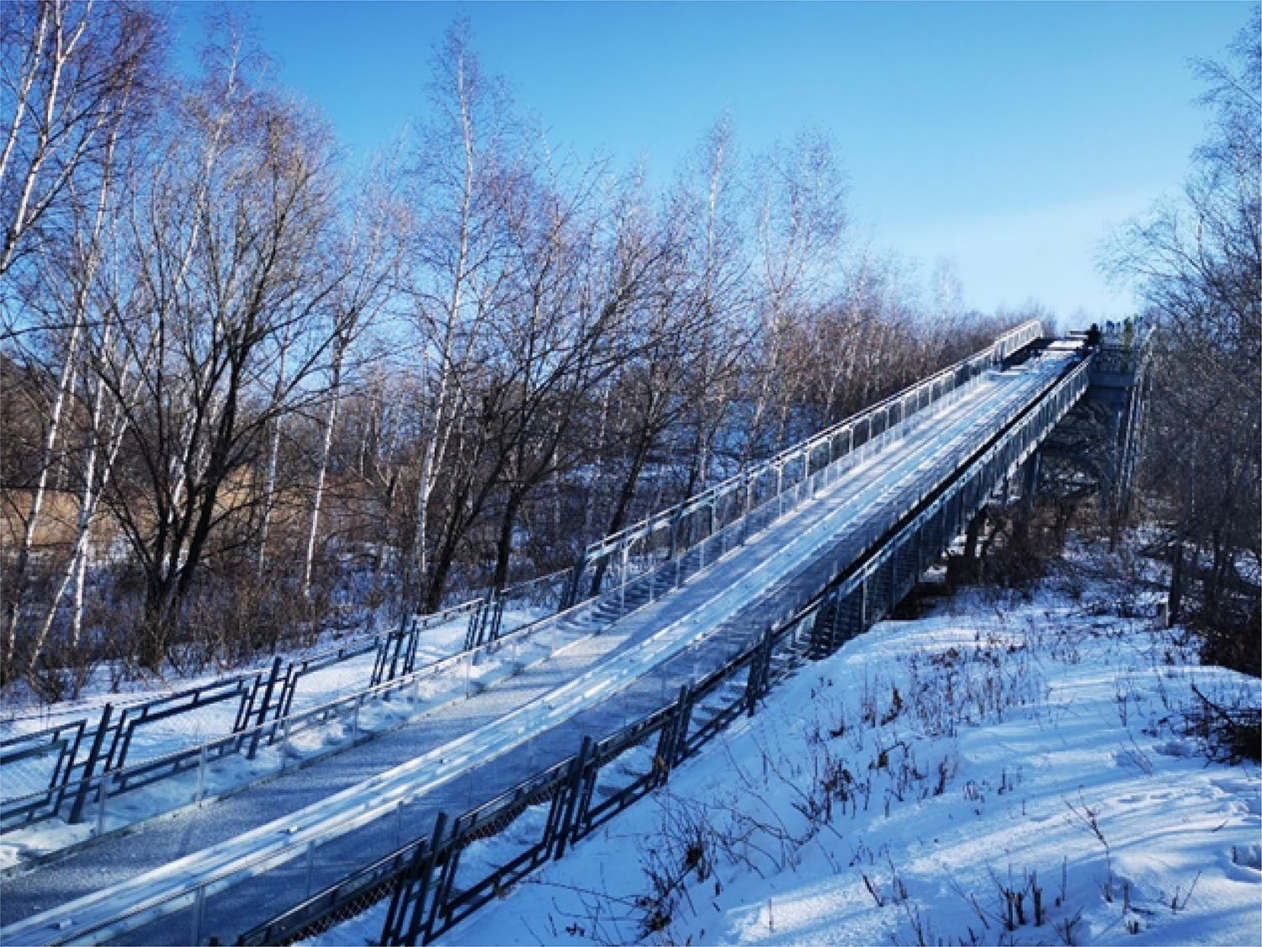
Panoramic view of the race track.

Commissioning of the ski jumping equipment took place in February, 2021, and the test was carried out in February, 2021. The collection site was the K50 ski jumping site of Jilin Beidahu Ski Resort. A panoramic view of the race track is shown in Fig. 1. The acquisition equipment included a Germany Simi Motion high-speed camera (with sampling frequency 200 Hz, fixed-focus shooting, and image resolution of 1200 × 800 pixels), and a Fastcam Mini WX100 high-speed camera (a small, lightweight, anti-vibration high-speed camera with a sampling frequency of 500 Hz, fixed-focus shooting, and image resolution of 2000 × 2000 pixels).

**Figure 2:**
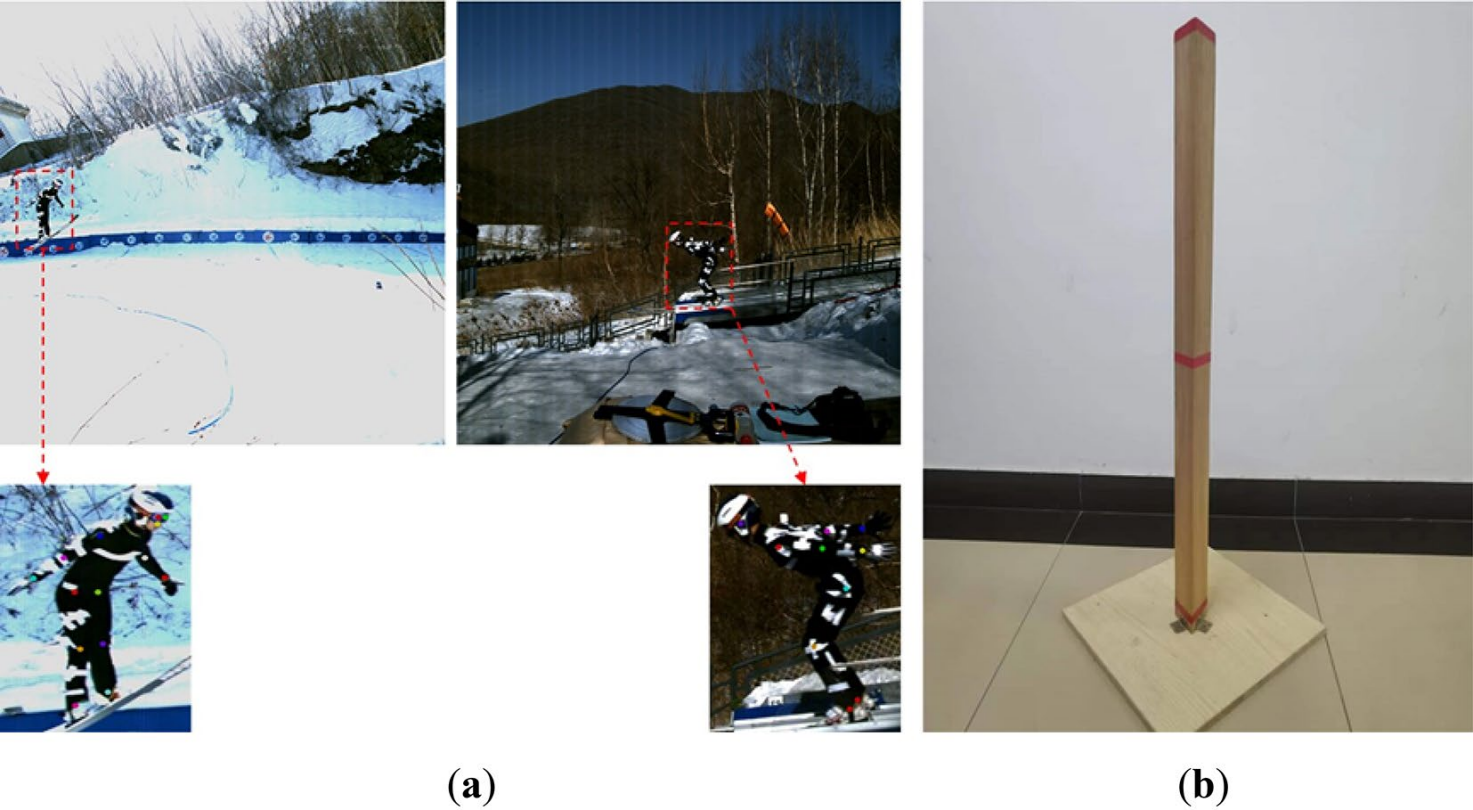
(**a**) Original image and local labeled keypoints of ski jumpers; (**b**) calibration bar.

In Fig. 2b, the actual length of the calibration bar from top to bottom is 1 m. We used the Labelme tool to label the top and bottom of the bar. To reduce the error, the labeling tool was used to label the two endpoints by partially enlarging them, and then the distance on the chart of the calibration bar could be calculated using the law of right triangles.

**Figure 3:**
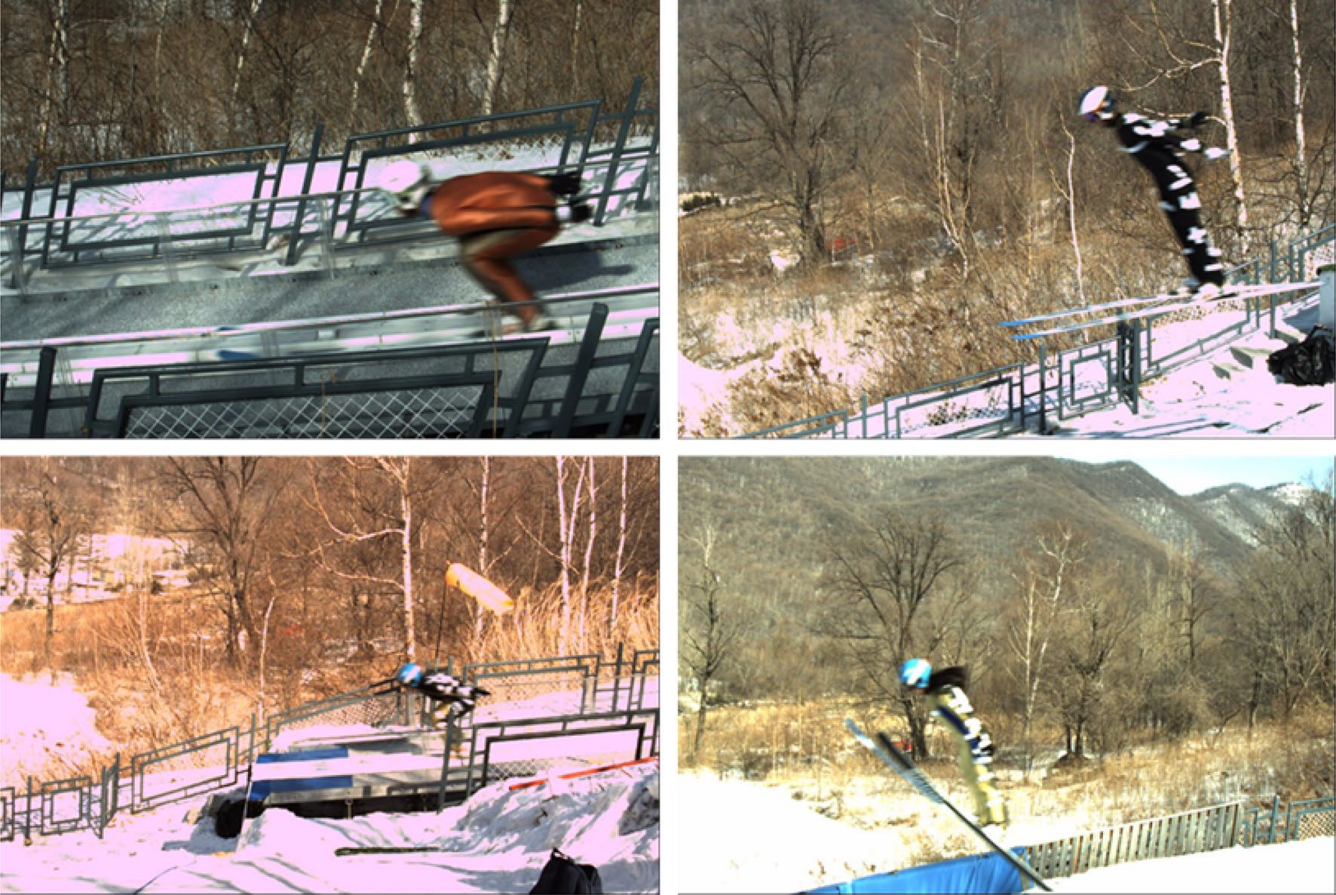
Examples of acquired images.

Video data were collected on five professional ski jumpers, including their name (referred to here as S1–S5), gender, years of experience, sporting level, height, weight and ski length. These five athletes performed a total of ten ski jumps (with two, three, three, one and one jumps for skiers S1 to S5, respectively), and the results were recorded. The motion video data on the ski jumpers were captured by the calibrated high-speed camera, and were converted into RGB image format for storage through Simi Motion software for frame-by-frame extraction. Examples of these images are shown in Fig. 3. The collected video data consisted of five phases of ski jumping (the in-run, take-off, early flight, stable flight and landing phases). We then constructed the keypoint dataset, called KDSJ. The data on athletes S1 to S3 in KDSJ were used to construct the training set, the data on athlete S4 were used to create the validation set, the data on athlete S5 were used to create the test set. There were 607, 315 and 200 images in the training set, validation set and test set, respectively. The visible keypoints and bounding boxes of the ski jumpers in the images were annotated using the Labelme (https://github.com/wkentaro/labelme, version: 4.5.13) under the guidance of experienced technicians. The original image and the locally labeled keypoints of the ski jumpers are shown in Fig. 2a. The important keypoints were the shoulder, elbow, wrist, hip, knee and ankle on both the left and right sides^6^. The labeled data format was made into the file format of the MS COCO 2017 dataset^30^ with the json format.

## Method

### HRNet architecture

**Figure 4:**
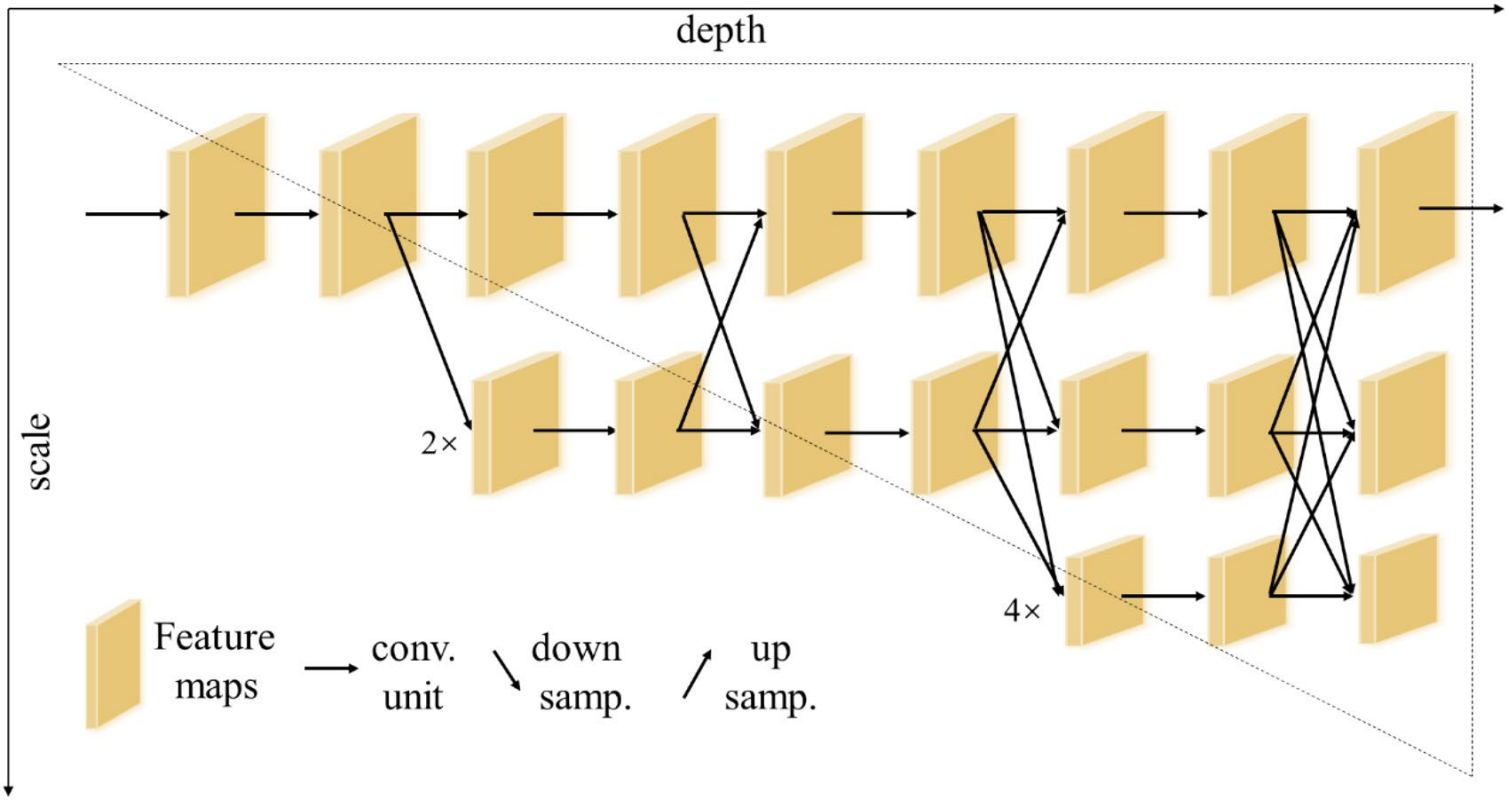
Architecture of the original HRNet^10^.

HRNet^10^ is a classical top-down method for human pose estimation that can maintain a high-resolution representation. The first stage contains high-resolution subnetworks, and the later stages gradually add subnetworks from high-to-low resolution. The multi-resolution subnetworks are connected in parallel. The parallel high-to-low resolution representations are repeatedly fused at multi-scales to obtain more high-resolution representations, so that the keypoint heatmap is predicted more accurately and is spatially more precise. The architecture of the original HRNet is shown in Fig. 4.

Compared with HRNet, the downsampling and upsampling operations used by methods such as Hourglass^7^, CPN^8^ and SimpleBaseline^9^ in the process of multi-scale feature extraction result in some feature information being lost. HRNet has two significant advantages. Firstly, the parallel connection method is used instead of the serial method to connect subnetworks from high-to-low resolution, which can maintain high resolution rather than recovering high resolution, and hence can reduce the loss of feature information and predict more accurate heatmaps. Secondly, by repeatedly performing multi-scale fusion, the high-resolution representation is enhanced by using the low-resolution representation of the same depth, to obtain a rich, high-resolution representation.

The high-speed motion of ski jumping causes motion blur, and it is therefore essential for HRNet to maintain high resolution for the accurate prediction of keypoints in images. In order to capture local cross-channel information and interact with local feature information of different channels, we propose a method called ECA-HRNet on the basis of HRNet for keypoint detection of ski jumpers.

### Architecture of ECA-HRNet

**Figure 5:**
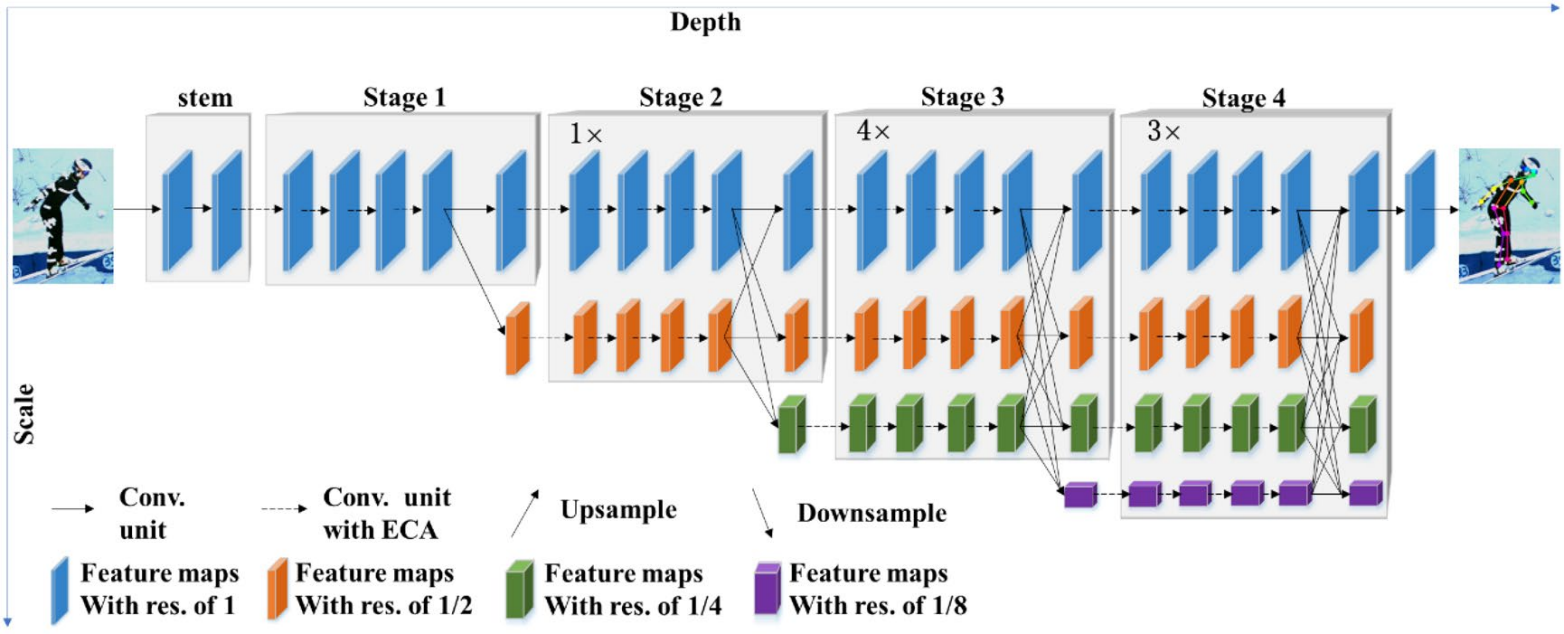
Architecture of ECA-HRNet.

The proposed ECA-HRNet is based on HRNet-W32, a lightweight backbone network in HRNet. The term “W32” represents the feature dimensions of the branch with the highest feature map resolution among the parallel branches. The feature dimensions of the other parallel branches are 64, 128 and 256, in that order. The architecture of ECA-HRNet is shown in Fig. 5.

ECA-HRNet starts with a stem consisting of two 3 × 3 convolutions with a step size of 1, in which the resolution of the feature map is reduced to 1/4 of the input image resolution. As shown in Fig. 5, the backbone network consists of four main stages. The first contains four residual units, each consisting of an ECA-Bottleneck (in which ECA module is embedded into a bottleneck) with a width of 64 and a 3 × 3 convolution to reduce the width of the feature map. The second (third, fourth) stage contains one (four, three) multi-resolution modules. Each multi-resolution module has two parts, consisting of parallel multi-resolution convolutions and multi-resolution fusion, as follows: (i) branches of different resolutions are connected in parallel, where each branch contains four residual units and each unit consists of an ECA-BasicBlock (where the ECA module is embedded into a BasicBlock); (ii) branches with different resolutions perform feature fusion to complete the exchange of information. Specifically, the transition from low-to-high resolution is achieved by bilinear upsampling, and from high to low by one or more cross-step convolutions (3 × 3 convolution layers with a step size of 2).

At the end of each stage (except the fourth), a 3 × 3 convolutional layer with a step size of 2 is applied to reduce the resolution, as the beginning of the new branch. Overall, the resolution is reduced by half (1, 1/2, 1/4, and 1/8) in each of the four branches from top to bottom, and the number of channels is doubled accordingly (32, 64, 128, and 256). Finally, a heatmap of 17 keypoints is obtained by a 1 × 1 convolution layer with a step size of 1.

### Efficient channel attention

Channel attention has brought significant improvements in the performance of deep convolutional neural networks^31^. Most of the existing methods are devoted to achieving more complex attention for better performance, which also increases the complexity of the model. To achieve a balance between performance and complexity, efficient channel attention (ECA)^29^ performs local cross-channel interaction without dimensionality reduction, which significantly reduces the model complexity while maintaining performance.

**Figure 6:**
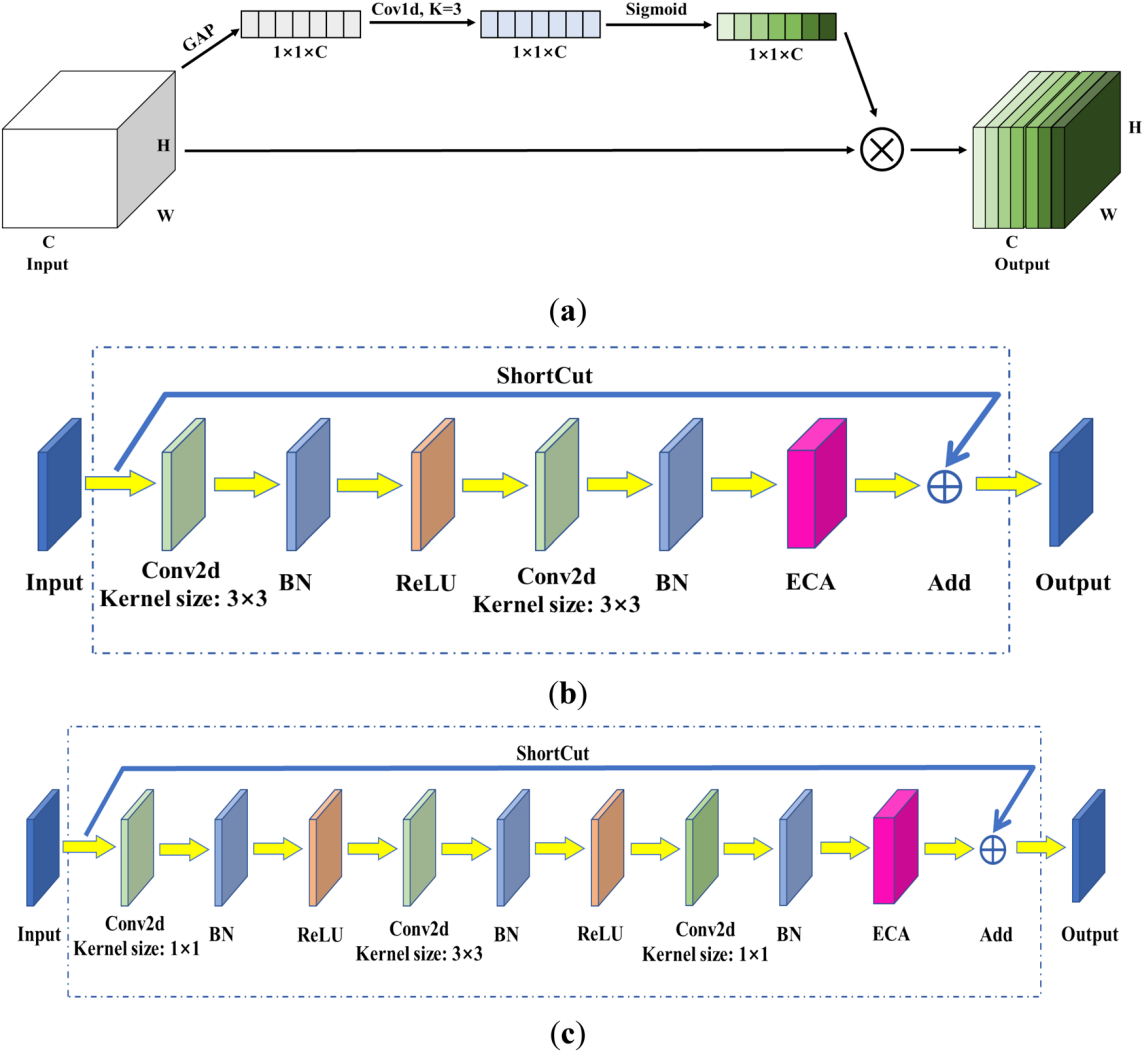
Structure of the ECA module and improved basic residual blocks: (**a**) ECA module; (**b**) ECA-BasicBlock; (**c**) ECA-Bottleneck.

The structure of the ECA module is shown in Fig. 6a. Since the size of the feature map input is $C\times H\times W$, it becomes $C\times 1\times 1$ after a global average pooling (GAP) layer. Because HRNet is a deep network and has many intermediate layers, which determine the feature extraction and fusion capability of the HRNet^10^, small convolution kernels can extract more features. Moreover, in pose estimation, the connectivity of human joints is related to the front and rear joints, but not to other joints. Therefore, the convolution kernel K of one-dimensional convolution is set to 3. The Sigmoid function are used to generate the corresponding channel weights, which represent the importance of each channel feature, the input features are weighted by multiplication to complete the recalibration of the features.

The deep convolutional neural network embedded with the ECA module is called ECA-Net, as shown in Fig. 6b,c. The output of the ECA module can be fed directly into the subsequent network layers. Bottleneck and BasicBlock are the classical convolutional units commonly used in ResNet. The ECA module embedded into the Bottleneck convolution unit is called ECA-Bottleneck, as shown in Fig. 6c, while the ECA module embedded into the BasicBlock convolution unit is called ECA-BasicBlock, as shown in Fig. 6b. Due to the simplicity of the ECA module, it can be directly embedded into existing network frameworks^29^.

### Training strategies

To further improve the precision of keypoint detection for ski jumpers, data augmentation and transfer learning were used in the training process. ECA-HRNet is a heavyweight network, and the convolutional layer of the feature extraction module needs to be fully trained to extract key features from images, which requires a large amount of data support. Hence, data enhancement with random rotation [− 45°,45°], random flipping, random scaling [0.65,1.35] and half-body data augmentation was used. When training a large network, the results obtained from feature knowledge transfer are better than those from randomly initialized network parameters. This method alleviates problems such as overfitting caused by insufficient data, speeds up the training, and improves the generalization ability of the model at the same time.

**Figure 7:**
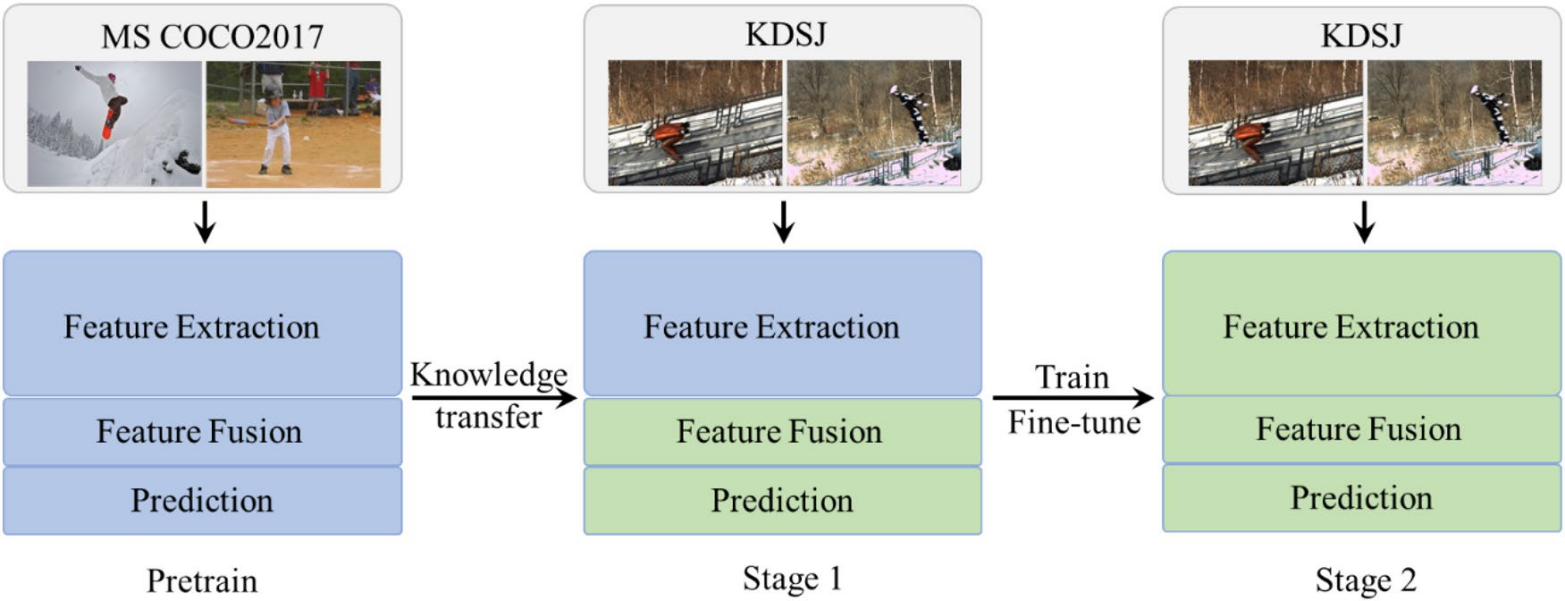
Transfer learning strategy.

At the training stage, MS COCO2017 was used as the source domain, and KDSJ as the target domain. First, ECA-HRNet was pre-trained on MS COCO2017, and the network parameters from the public dataset were then used to initialize ECA-HRNet to realize the transfer of feature knowledge. Finally, the hyperparameters of the network were dynamically fine-tuned in the target domain dataset to obtain a better keypoint detection model. The transfer learning strategy is illustrated in Fig. 7.

### Method framework

**Figure 8:**
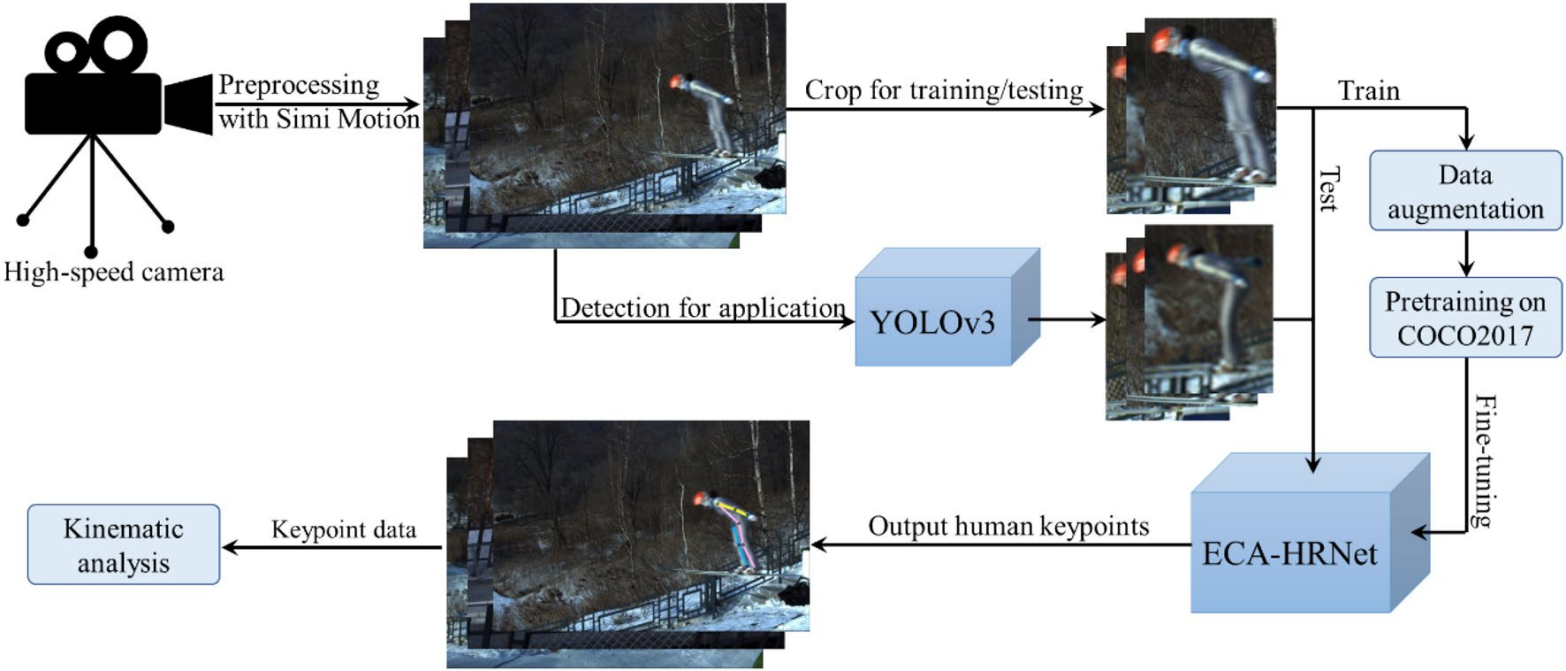
Schematic framework of the ski jumper pose estimation.

Figure 8 shows a schematic of the framework for the ski jumper pose estimation method. The main steps in the framework are image preprocessing, training and testing of the ECA-HRNet, and motion analysis. We used a high-speed camera to capture videos in a complex environment, and converted them into frame images. We then constructed a keypoint dataset of ski jumpers named KDSJ. ECA-HRNet was trained using a transfer learning method, and the final model obtained after fine-tuning was used as our ski jumper keypoint detection model. The specific steps in the ski jumper estimation method are as follows:

1. Videos of the ski jumpers’ training process were captured by high-speed cameras, and then converted into frames which were used to construct a keypoint dataset of ski jumpers, called KDSJ.
2. The images in KDSJ were divided into training set, validation set, test set in a 6:3:2 ratio.
3. Images were annotated using Labelme, and annotation information such as the bounding boxes and visible keypoints of the ski jumpers were saved as JSON files.
4. ECA-HRNet was constructed, and pre-trained on MS COCO2017 to acquire the initial parameters for feature knowledge transfer.
5. The ECA-HRNet parameters were fine-tuned on KDSJ to obtain the best model in terms of precision.
6. The application process of the ECA-HRNet was as follows: each frame of the live video was acquired in real time; the position of the athlete in each continuous frame image was detected using the YOLOv3 object detection method; the keypoints of the athletes were predicted using the trained ECA-HRNet model to achieve batch processing of the keypoint prediction; and finally, the keypoint data were saved.
7. The kinematic parameters of the athletes (using joint angles over time as an example) were analyzed using the keypoint data from the continuous frames.

### Statement

Written informed consent was obtained and agreed to by all participants before the manuscript was submitted. All participants agree to publish identifying information/images in an online open access publication.

## Experimental results and analysis

### Experimental environment and parameters

The configuration of the hardware and software environments used in our experiments is shown in Table 1.

**Table 1:** Configuration of the hardware and software environments.

Platform	Configuration
Operating system	Ubuntu18.04 LTS 64-bits
CPU	Intel(R) Core (TM) i7-9700
GPU	NVIDIA GeForce RTX 2070Ti
GPU accelerator	CUDA 10.2 and cuDNN 7.6.5
Deep learning frame	PyTorch1.2
Compilers	PyCharm and Anaconda
Scripting language	Python 3.6

The learning rate of ECA-HRNet was adjusted during the training process by setting it to 1 × 10^–4^ and 1 × 10^–5^ at the 170th and 200th epochs, respectively. The mean square error (MSE) loss function was used, as shown in Eq. (1). The other training parameters are shown in Table 2.

1 $$MSE=\frac{1}{N}\sum_{1}^{N} ({x}_{i}-{{x}_{i}}^{^{\prime}}{)}^{2}$$

where $N$ is the number of ground truth keypoints for each person instance, ${{x}_{i}}^{^{\prime}}$ is the heatmap of predicted keypoints, and ${x}_{i}$ is the ground truth heatmap.

**Table 2:** Configuration of the training parameters.

Parameter	Value
Input size	$256\times 192\times 3$
Optimization algorithm	Adam
Batch size	32
Training epochs	210
Base learning rate	0.001
Momentum	0.9
Weight decay	0.0001

### Evaluation metric

In the human keypoint detection task, the standard evaluation metric was based on object keypoint similarity (OKS), defined as shown in Eq. (2):

2 $$OKS=\frac{\sum_{i} exp(-{d}_{i}^{2}/2{s}^{2}{k}_{i}^{2})\delta ({v}_{i}>0)}{\sum_{i} \delta ({v}_{i}>0)}$$

where ${d}_{i}$ is the Euclidean distance between the detected keypoint and the corresponding ground truth, $s$ is the object scale; ${v}_{i}$ is the visibility flag of the ground truth (where '0' means the keypoint is not visible and not labeled, '1' means the keypoint is labeled but not visible, and '2' means the keypoint is both labeled and visible), and ${k}_{i}$ is a per-keypoint constant that controls the falloff.

Standard evaluation metrics used in the keypoint detection experiment included the average precision (AP) and average recall (AR) scores. AP was calculated as the mean score for 10 positions ($OKS=$ 0.50,0.55, …, 0.90,0.95), and AR was also defined as the mean score for 10 positions ($OKS=$ 0.50,0.55, …, 0.90,0.95).

### Validation results

**Figure 9:**
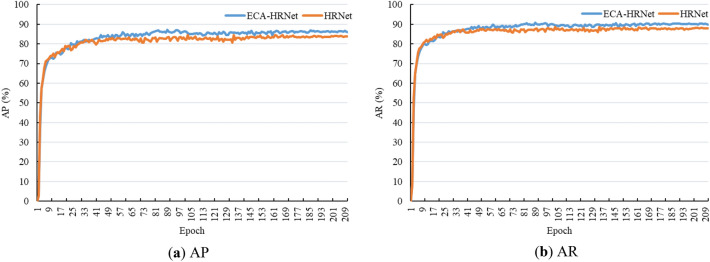
AP and AR curves for HRNet and ECA-HRNet.

The AP and AR curves for HRNet and ECA-HRNet, calculated for the validation set of KDSJ for training over 210 epochs with both transfer learning strategies, are shown in Fig. 9a,b respectively. It can be seen from the figures that the AP and AR curves gradually tend towards smoothness. Both the AP and AR curves of ECA-HRNet were higher than those of HRNet. Hence, the ECA-HRNet model after 210 epochs was used as our keypoint detection model for ski jumpers.

### Comparison of experimental results

#### Comparison of experimental results on public dataset

COCO2017^30^ contains more than 200 K images and 250 K person instances and is annotated with 17 keypoints. The training set used was the COCO train2017 dataset, which contained 57 K images and 150 K person instances. The validation set used was the val2017 dataset, which consisted of 5 K images.

To verify the effectiveness of the proposed ECA-HRNet method, we compared it with several mainstream pose estimation methods, such as the 8-stage Hourglass^7^, CPN^8^, SimpleBaseline^9^, and HRNet^10^. These mainstream methods were trained on the COCO2017 dataset, and the lightweight network framework HRNet-W32 was used in the experiments. The hyperparameter settings, input size (256 × 192), learning rate strategy and the number of training epochs were the same as those in^10^. Under the hardware and software conditions given in Table 1 and the parameter settings listed in Table 2, we trained the model on a single 8G NVIDIA GeForce RTX 2070Ti GPU for about 5 days. We used the same Faster RCNN as in^10^, and some of the experimental results cited in^10^.

##### Results on COCO2017 validation set

Table 3 shows a comparison of the results with those of other human pose estimation methods on the COCO2017 validation set. The proposed ECA-HRNet, which outperformed the other classical human pose estimation methods with the same input size, was trained from scratch and achieved an AP score of 74.4%. Several points should be noted:

i. Compared to the HRNet in^10^, the HRNet we trained achieved the same AP and the same number of parameters (Params), but with a 0.2% improvement in AR and a slight increase in GFLOPs (only 0.01 GFLOPs).
ii. Compared with HRNet trained by us, the proposed ECA-HRNet showed a slight increase in the number of parameters and GFLOPs (3.24 × 10^–4^ M and 3.0 × 10^–5^ GFLOPs, respectively). Most importantly, the AP and AR of the proposed ECA-HRNet showed large improvements of 1.0% and 0.7%, respectively.

**Table 3:** Comparison on the COCO2017 validation set (Pretra = backbone pretrained on the ImageNet classification task; OHKM = online hard keypoint mining^8^).

Method	Backbone	Pretra	Params	GFLOPs	AP	AR
8-stage Hourglass^7^	8-stage Hourglass	N	25.1 M	14.3	66.9	–
CPN + OHKM^8^	ResNet-50	Y	27.0 M	6.20	69.4	–
SimpleBaseline^9^	ResNet-50	Y	34.0 M	8.90	70.4	76.3
SimpleBaseline^9^	ResNet-101	Y	53.0 M	12.4	71.4	77.1
SimpleBaseline^9^	ResNet-152	Y	68.6 M	15.7	72.0	77.8
HRNet^10^	HRNet-W32	N	28.5 M	7.10	73.4	78.9
HRNet (our implement)	HRNet-W32	N	28.5 M	7.11	73.4	79.1
ECA-HRNet (our)	HRNet-W32 + ECA	N	28.5 M	7.11	74.4	79.8

**Figure 10:**
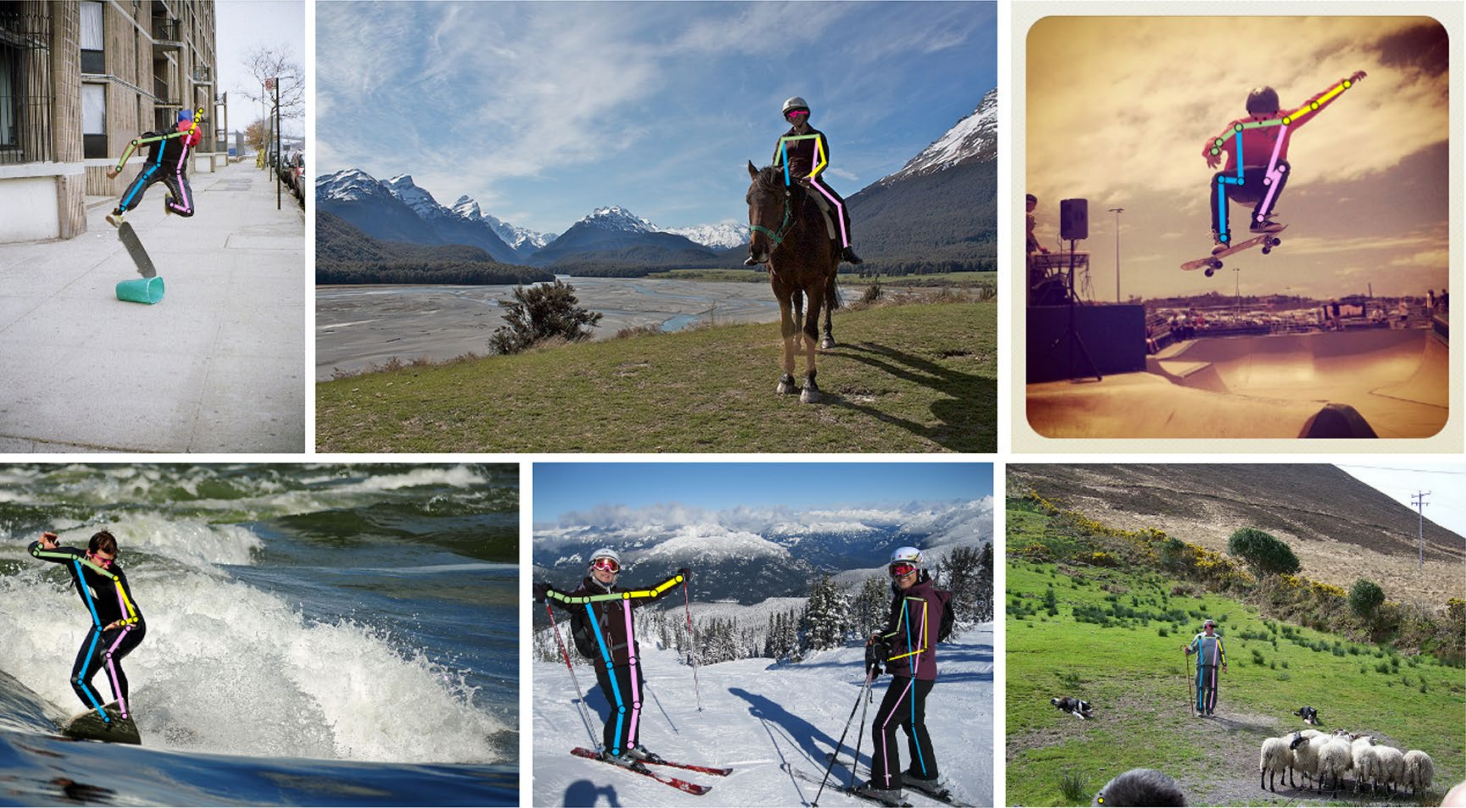
Results from the proposed ECA-HRNet model on the COCO2017 validation set.

Figure 10 shows a visualization of the detection results for the proposed ECA-HRNet on the COCO2017 validation set.

##### Results on COCO2017 test-dev set

Table 4 shows a comparison of the results with those of several mainstream human pose estimation methods on the COCO2017 test-dev set. The input size used in the experiment of the method proposed in this study is 256 × 192, which is different from the input size 384 × 288 in^10^. Because the latter requires larger training resources and the model obtained after training is larger, the calculation speed will be greatly reduced. The proposed ECA-HRNet, was trained from scratch and achieved an AP score of 73.4%, which outperformed the mainstream human pose estimation methods. In order to maintain the fairness of comparison, the test results given by HRNet are also trained from scratch. The proposed ECA-HRNet received 0.6 improvements compared with HRNet in AP and AR, respectively.

**Table 4:** Comparisons on the COCO test-dev set. #Params and FLOPs are calculated for the pose estimation network, and those for human detection are not included.

Method	Backbone	Input size	Params	GFLOPs	AP	AR
Mask-RCNN^24^	ResNet-50-FPN	–	–	–	63.1	–
CPN^8^	ResNet-Inception	384 × 288	–	–	72.1	78.5
RMPE^32^	PyraNet	320 × 256	28.1 M	26.7	72.3	–
HRNet^10^	HRNet-W32	256 × 192	28.5 M	7.10	72.8	78.3
ECA-HRNet (our)	HRNet-W32 + ECA	256 × 192	28.5 M	7.11	73.4	78.9

#### Comparison of experimental results on the ski jumping dataset

To further validate the effectiveness of the proposed ECA-HRNet for ski jumper pose estimation, the experimental results were compared with those of the mainstream human pose estimation methods, i.e., the 8-stage Hourglass^7^, CPN^8^, SimpleBaseline^9^, and HRNet^10^, under transfer learning. The results were based on the ground truth bounding box and the same input size (256 × 192).

##### Results on KDSJ validation set

**Figure 11:**
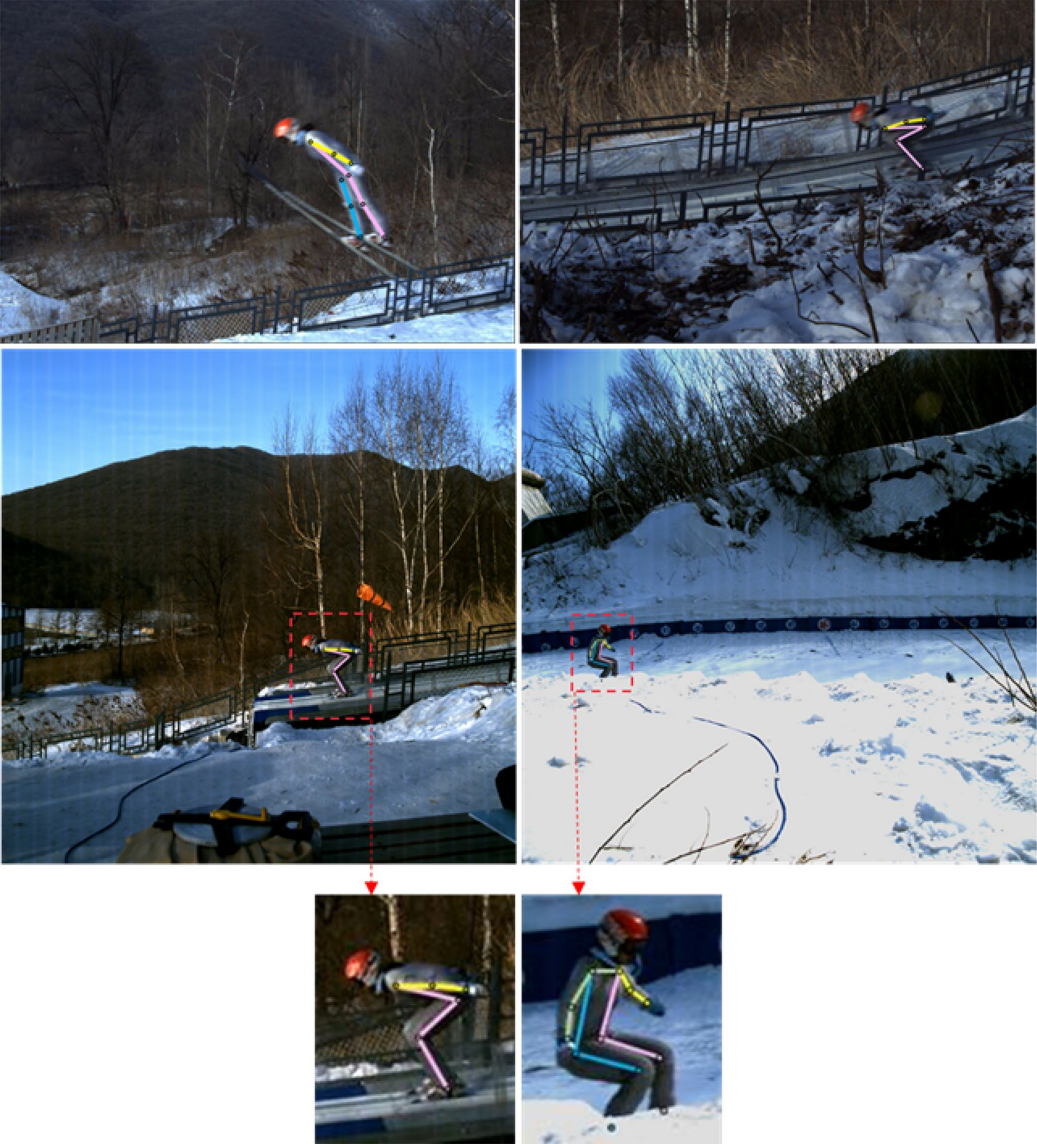
Visualization of results on the KDSJ validation set.

The proposed ECA-HRNet achieved an AP score of 87.1%, which was better than the other classical human pose estimation methods for the same input size as shown in Table 5. Figure 11 shows the results from the proposed ECA-HRNet on the KDSJ validation set. We note the following:

i. Compared to Hourglass, the AP score of the proposed ECA-HRNet showed an improvement of 39.3%.
ii. Compared to CPN, the proposed ECA-HRNet obtained a significant improvement of about 16 points.
iii. The proposed ECA-HRNet achieved an increase in the AP score of 5.7% compared to the SimpleBaseline model with the ResNet-50 backbone, and as shown in Table 5, the difference between these two methods in terms of the number of parameters and GFLOPs was very small. Compared to the SimpleBaseline with the ResNet-152 backbone, the AP score was increased by 3.0%, and as shown in Table 5, the numbers of parameters and GFLOPs were half as large.
iv. Compared to HRNet, the AP score of the proposed ECA-HRNet was improved by 2.5%, with almost no increase in the number of parameters and GFLOPs.

**Table 5:** Comparison of results on KDSJ validation set (Pretra = backbone pretrained on the COCO2017 keypoint task; OHKM = online hard keypoint mining^8^).

Method	Backbone	Pretra	AP	AR
8-stage Hourglass	8-stage Hourglass	Y	47.8	–
CPN + OHKM	ResNet-50	Y	71.0	–
Mask-RCNN	ResNet-50-FPN	Y	78.6	–
SimpleBaseline	ResNet-50	Y	81.4	85.5
SimpleBaseline	ResNet-101	Y	82.4	86.5
SimpleBaseline	ResNet-152	Y	84.1	87.3
HRNet	HRNet-W32	Y	84.6	88.3
ECA-HRNet (our)	HRNet-W32 + ECA	Y	87.1	90.7

##### Results on KDSJ test set

Table 6 shows a comparison of the results with those of several mainstream human pose estimation methods on the KDSJ test set. The proposed ECA-HRNet achieved an AP score of 86.4%, which was better than the other classical human pose estimation methods for the same input size. Compared to the HRNet with the same input size, the proposed ECA-HRNet received 2.8 and 2.4 improvements in AP and AR, respectively.

**Table 6:** Comparison of results on KDSJ test set (Pretra = backbone pretrained on the COCO2017 keypoint task; OHKM = online hard keypoint mining^8^).

Method	Backbone	Pretra	AP	AR
8-stage Hourglass	8-stage Hourglass	Y	46.9	–
CPN + OHKM	ResNet-50	Y	70.1	–
Mask-RCNN	ResNet-50-FPN	Y	77.9	–
SimpleBaseline	ResNet-50	Y	80.7	83.5
SimpleBaseline	ResNet-101	Y	81.5	83.9
SimpleBaseline	ResNet-152	Y	83.2	84.9
HRNet	HRNet-W32	Y	83.6	86.1
ECA-HRNet (our)	HRNet-W32 + ECA	Y	86.4	88.5

Due to the parallel connections between networks and the fusion of repetitive multi-scale feature information, HRNet and the proposed ECA-HRNet methods can maintain high resolution^10^. From Table 6, compared to other mainstream methods, both of these two methods achieved higher accuracy for ski jumpers in blurred images compared to other mainstream methods. In addition, the proposed ECA-HRNet, which fuses more cross-channel feature information, had higher AP and AR scores. Hence, the proposed ECA-HRNet model was better than HRNet and was more suitable for keypoint detection of ski jumpers.

## Ski jumping motion analysis

Most current research has focused on the take-off^33–35^and early flight phases^36–38^, as these are considered the most critical of the five phases in terms of their impact on performance^35,39^. In this study, the kinematic characteristics of the take-off and early flight phases for the athlete S4 (capture frequency 500 Hz, resolution 2000 × 2000 pixels) were analyzed using the keypoint information detected by the proposed ECA-HRNet. When using 2D images, the evaluation of the ski jumping motion mainly occurs in the sagittal plane^4^, which can be analyzed on the athlete's one side based on the position of the high-speed camera.

**Figure 12:**
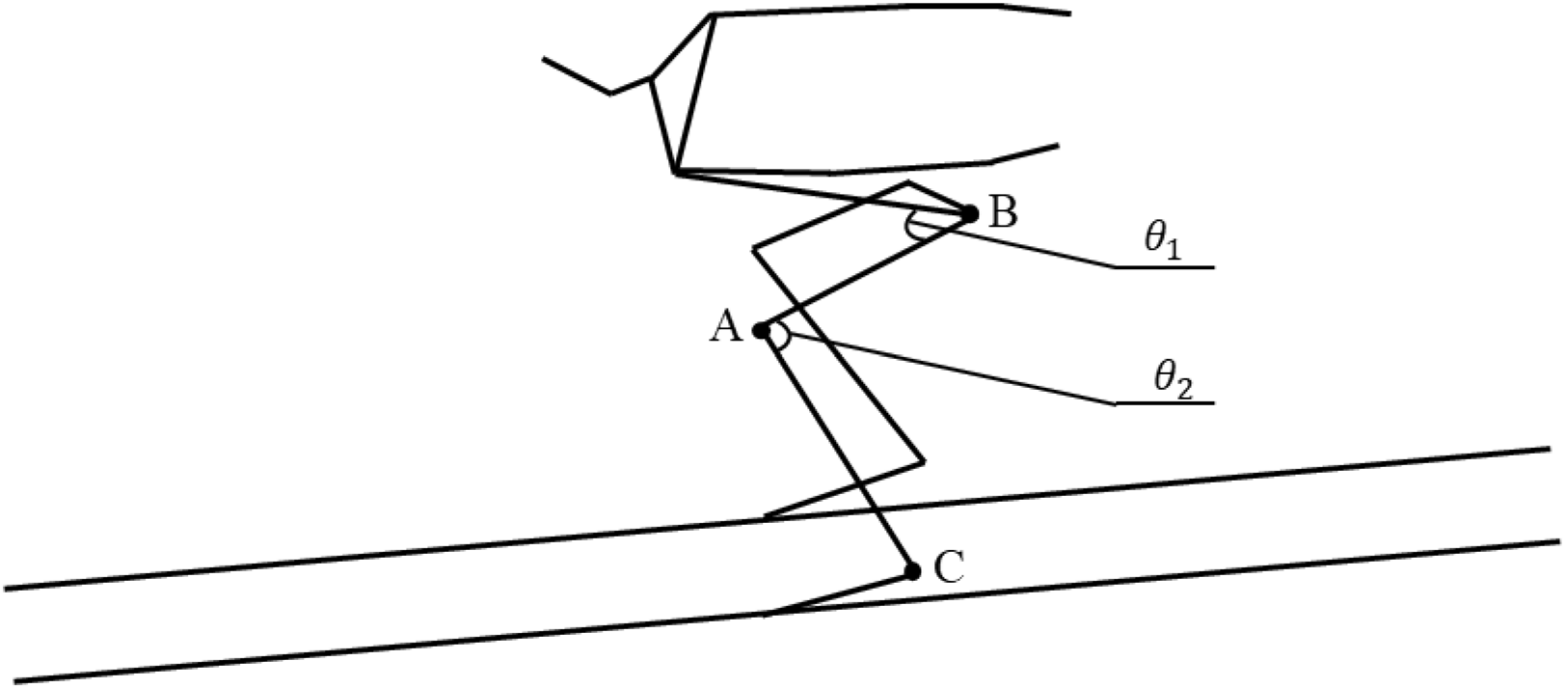
Diagram of the angles at the hip and knee.

When assessing ski jumping technique, the hip and knee joints play an important role in generating power in the two most critical phases^35^. A graph of variation in joint angle with the number of frames was calculated from the keypoint data detected by the proposed ECA-HRNet. In this graph, the number of consecutive frames is used as the horizontal axis (X-axis) and the joint angle as the vertical axis (Y-axis). The calculated angle was smoothed with a fourth-order Butterworth low-pass filter with a cutoff frequency of 3 Hz^25,40^. The hip angle (${\theta }_{1}$ in Fig. 12) is defined as the anterior angle between the trunk and the thigh, whereas the knee angle (${\theta }_{2}$ in Fig. 12) is defined as the angle between the thigh and the calf, as shown in Fig. 12. The angle vector through the keypoints is calculated as shown in Eq. (3):

3 $${\theta }_{i}=arccos\frac{{\varvec{A}}{\varvec{B}}\cdot {\varvec{A}}{\varvec{C}}}{\left|{\varvec{A}}{\varvec{B}}\right| |{\varvec{A}}{\varvec{C}}|}$$

where *i* denotes the hip or knee keypoint. The three keypoints are denoted as $A({x}_{1},{y}_{1})$, $B({x}_{2},{y}_{2})$ and $C({x}_{3},{y}_{3})$, respectively. Keypoint $A$ is the hip or knee, ${\theta }_{1}$ is the angle made by the shoulder, hip and knee. ${\theta }_{2}$ is the angle made by the hip, knee and ankle.

**Figure 13:**
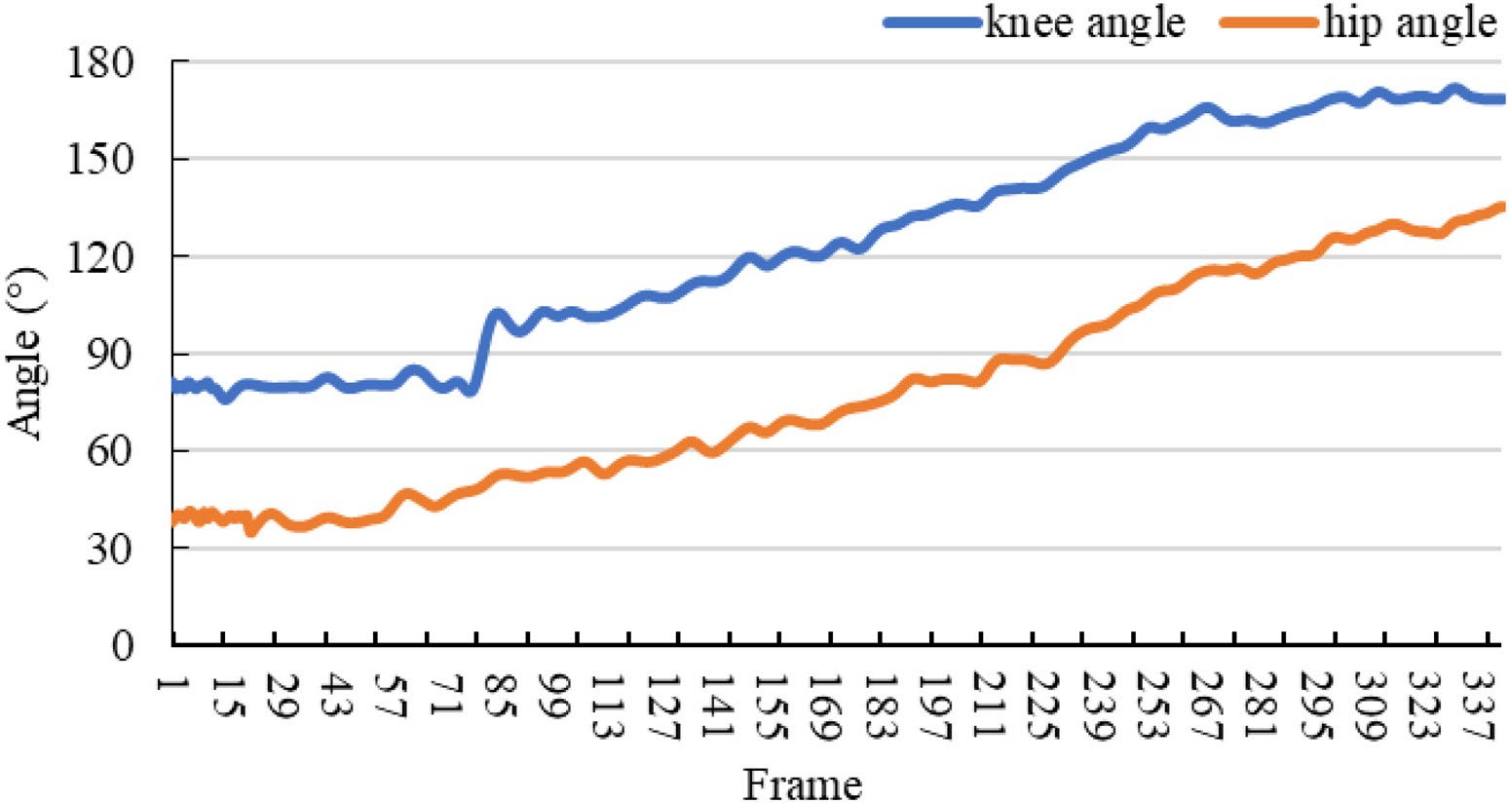
Changes in hip/knee angle versus frame number.

The trends calculated for the hip and knee angles of athlete S4 versus the number of frames are shown in Fig. 13.

From the overall changes in joint angles shown in Fig. 13, athlete S4 maintains the in-run phase pose before take-off, so ${\theta }_{1}$ and ${\theta }_{2}$ remain almost constant, with both being similar to the horizontal line. After entering the take-off phase, the body pose is rapidly extended. Thus, both angles tend to rise, with the hip angle starting to rise earlier than the knee angle. The hip and knee angles continue to increase during the early flight phase. Unlike the hip joint, the knee angle tends to gradually stabilize in the later stages of early flight, while the hip angle continues to extend.

From the local changes in the joint angles in Fig. 13, we note the following:

1. During take-off, the change in vertical velocity is proportional to the vertical force^35^, and since the vertical position of the athlete at the end of the jump has a substantial effect on the initial conditions of the flight, this should enable the maximum vertical velocity to be achieved by maximizing the jumping force perpendicular to the jumping platform^36^. Furthermore, the amount of jumping force can be maximized by extending the knee joint at maximum speed during the jump. During the take-off by the athlete S4 in Fig. 13, both the knee and hip angles decrease and then increase. The point of descent of the knee is in frame 65, and the ascending point (the lowest point of the squat) is in frame 78, the point of descent of the hip is in frame 64, while the ascending point is in frame 69. The angles of the two joints are reduced by about 6° and 3°, respectively, and we therefore suggest training to improve the explosive power of the knee joint, such as squatting, jumping etc.
2. The take-off characteristics are mainly divided into two categories: one involves extending the knee angle first and then the hip angle, whereas the other involves extending the hip angle first and then the knee angle. Wind tunnel experiments have demonstrated that the increase in the air drag coefficient due to the hip angle is much larger than that due to the knee angle, meaning that the former take-off style is better than the latter. Figure 13 shows that the take-off of athlete S4 is characterized by first hip extension and then knee extension. This method increases the air resistance during the take-off and early flight phases, and we therefore recommend using the correct style of jumping, with knee extension first and then hip extension.
3. The main challenge in the early flight phase is to adjust the pose as quickly as possible to achieve stable flight. In this phase, athlete S4 rapidly extended the hip and knee angles within a very short time, reaching an approximate hip angle of 160°^41^and extending the knee joint as far as possible. From Fig. 13, it can be seen that the knee angle of athlete S4 tends to stabilize at about frame 300, reaching about 168°. Measured from the start of the jump, the hip extension lasts longer, and we therefore recommend increasing the speed of the hip extension.

## Conclusions

In recent years, interest of people in skiing has increased. As one of the events of the Winter Olympics, ski jumping has also received wide attention. However, since it is a high-speed sport, people find it difficult to analyze the motion of a ski jumper in a subjective way. To solve this problem, we combined the use of an ECA module with transfer learning to improve the HRNet. We proposed our ECA-HRNet for keypoint detection of ski jumpers and analyzed the motions involved in ski jumping.

First, video data were acquired from a calibrated high-speed camera and converted into images to construct a keypoint detection dataset called KDSJ, which was then divided into training set, validation set and test set in a 6:3:2 ratio. Next, ECA was embedded in the multi-scale feature extraction module of HRNet to enhance the interaction of feature information across channels and to improve the network feature extraction capability. The accuracy of keypoint detection was improved with the AP achieving the average precision of 73.4% on COCO2017 test-dev set, which was higher than for the original HRNet. The feature knowledge from the public dataset was transferred to the task of ski jumping via transfer learning. The proposed ECA-HRNet outperformed other mainstream human pose estimation methods by achieving an AP of 86.4% under the ground truth bounding box on the test set of KDSJ. Transfer learning was also used to improve the generalization ability of the model, avoid overfitting, and speed up the training of the model. Finally, YOLOv3 was used as the object detector to detect ski jumpers in images and the proposed ECA-HRNet model was applied to estimate the keypoints of the ski jumpers. For athlete S4, we analyzed the changes in the angles at the knee and hip joints over the frames of the in-run and early flight phases and training recommendations were made.

The proposed ECA-HRNet only deals with ski jumping data and is therefore only applicable to this single sport. In future work, we will add captured freestyle skiing and snowboarding data to increase the generalization capability of the proposed ECA-HRNet in order to allow it to be applied to more winter sports.

## Data Availability

The dataset of ski jumpers generated during the current study are available from the corresponding author on reasonable request.
